# Parental Socialization and Its Impact across the Lifespan

**DOI:** 10.3390/bs10060101

**Published:** 2020-06-16

**Authors:** Jose Antonio Martinez-Escudero, Sonia Villarejo, Oscar F. Garcia, Fernando Garcia

**Affiliations:** 1 Department of Methodology of Behavioral Sciences, Faculty of Psychology, University of Valencia, 46010 Valencia, Spain; jomares2@alumni.uv.es (J.A.M.-E.); sovite@alumni.uv.es (S.V.); fernando.garcia@uv.es (F.G.); 2 Department of Developmental and Educational Psychology, Faculty of Psychology, University of Valencia, 46010 Valencia, Spain

**Keywords:** parental socialization, adolescence, adult development

## Abstract

Classical studies have found that parental warmth combined with parental strictness is the best parental strategy to promote children’s psychosocial development. Nevertheless, a growing set of emergent studies has questioned the benefits of parental strictness. The present study examined parental socialization and its short- and long-term impact on the psychosocial development of adolescents and adult children. The sample consisted of 2150 Spanish participants, 623 adolescents (12–18 years), 619 young adults (19–35 years), 502 middle-aged adults (35–59 years), and 406 older adults (60 years or older). Families were classified into one of four typologies (indulgent, authoritative, authoritarian, and neglectful). Psychosocial development was examined with five indicators (physical and family self-concept, nervousness, empathy, and internalization of social values of benevolence). The results show a common short- and long-term pattern between parenting styles and psychosocial development: the indulgent style equaled or even surpassed the authoritative style, whereas the neglectful and authoritarian styles were associated with low scores. The present findings were discussed by considering the importance of the cultural context in family socialization. Additionally, the long-term impact of parental socialization seems to be crucial, even in adulthood.

## 1. Introduction

Parental socialization consists of parents’ influence on their children, in order to, among other objectives, encourage them to learn to inhibit actions that may be annoying or harmful to others and, at the same time, acquire behaviors that society demands, including consideration for others, self-reliance, accepting responsibility, and skills that will help them to become competent adults [1]. As socialization theorists explain, modern societies cannot rely on the ubiquitous, permanent, and indefinite presence of police or supervisors (for example, parents or caregivers) to ensure that individuals conform to social norms [2,3]. For this reason, the parents’ own socializing actions must lead the child to a certain degree of self-regulation (i.e., self-control) with regard to social norms. There comes a time when parental socialization ends: the teenager is now an adult. Unfortunately, not all children reach the socialization goals and become responsible adult members of their society [4,5].

**Figure 1:**
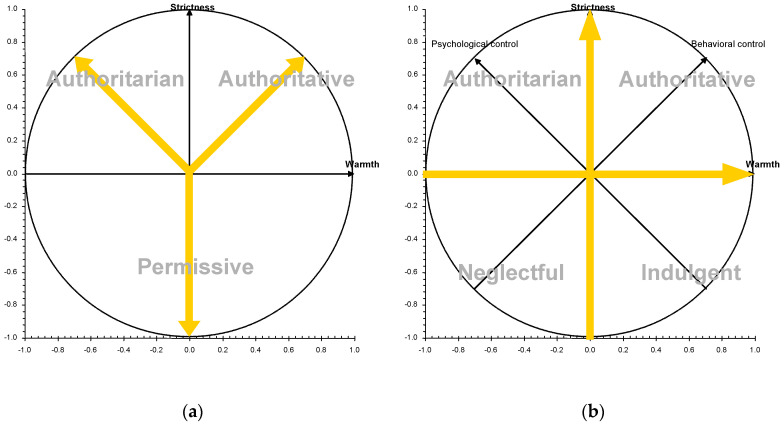
(**a**) Theoretical tripartite model ‘Y’; Representation of the tripartite model ‘Y’ proposed by Diana Baumrind [7]. (**b**) Theoretical fourfold model based on the two orthogonal dimensions; Representation of the fourfold model in which parenting practices and styles are examined within the two orthogonal dimensions [1,11,14,15,16].

In the study of parental socialization, Diana Baumrind defined a tripartite model based on the interaction between affection, communication, and control, yielding three parenting styles: authoritative, authoritarian, and permissive [6]; these three parenting patterns corresponded to three modes of parental control, the authoritative control, the authoritarian control, and the lack of control (i.e., permissive control) [7,8] (see Figure 1). However, the theoretical framework proposed by Maccoby and Martin (1983) [1] became the referential model for the study of parental socialization using two theoretically orthogonal axes, warmth (also called responsiveness, acceptance, or affection) and strictness (also called demandingness or imposition), and four styles [9,10,11,12,13].

It is important to note that scholars for these two orthogonal parental dimensions used different labels, although their operationalization was very similar. Historically, the warmth dimension has been labeled by early scholars as acceptance [17], assurance [18], nurturance [19,20], warmth [21,22], or love [14]; all of these labels have a similar meanings to acceptance/involvement [15]. The strictness dimensions has also been labeled as domination [17], hostility [18], inflexibility [22], control [14,23], firm control [24] or restriction [21]; all of these labels have a similar meanings to strictness/supervision [15]. In this sense, although schoolars have worked from different theoretical perspectives (e.g., behaviorist and Freudian theory), they described a simmilar parenting patterns [3,11,25]. As Steinberg noted (2005, p. 71), “responsiveness was often operationalized using measures of parental warmth and acceptance, while demandingness came to be defined with respect to parental firmness" [24]. The four parenting styles are defined by combining the two orthogonal dimensions: authoritative parents (high warmth and high strictness), authoritarian parents (low warmth and high strictness), indulgent parents (high warmth and low strictness), and neglectful parents (low warmth and low strictness) [26,27,28]. The empirical validation of the Maccoby and Martin model (1983) [1] with a sample of more than 10,000 adolescents and their families [15] showed the need to distinguish between two types of "permissive" parents: indulgent (with warmth) and neglectful (without warmth).

**Figure 2:**
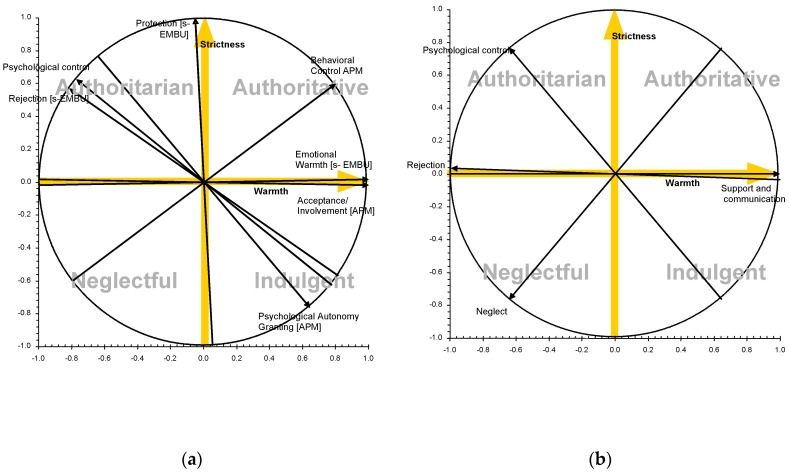
Representation of the fourfold model in which parenting practices and styles are examined within the two orthogonal dimensions in the Spanish cultural context. (**a**) Garcia et al., 2015; Empirical study of Garcia et al., 2015 [32]. (**b**) Tur-Porcar et al., 2019; Empirical study of Tur-Porcar et al., 2019 [33]. *Note.* ^[APM]^ The Authoritative Parenting Measure, APM [15,38]. ^[BAR]^ The Psychological Control Scale – Youth Self-Report, PCS-YSR [39]. ^[s-EMBU]^ The S(hort)-EMBU [40,41,42]. The measures of Tur-Porcar et al. 2019 are from Child Reports of Parental Behavior Inventory, CRPBI [43,44].

The fourfold theoretical parenting model (i.e., authoritative, indulgent, authoritarian and neglectful) emphasizes the need to examine the combined effects of the two parental dimensions (i.e., warmth and strictness) in order to accurately analyze the relationship between parental socialization and child adjustment [15]. The two parental dimensions would express two types of persistent patterns in parental strategies, theoretically defined as independent or orthogonal constructs (they are not related, and parenting in one of them does not allow us to know what parenting will be like in the other). Furthermore, the fourfold theoretical parenting model also helps researchers to organize different parenting practices according to these parental dimensions [1,11] (see Figure 1). For example, parenting practices based on psychological control are characterized by high strictness and low warmth, which are related to authoritarian parents [29]; parenting practices of behavioral control are characterized by high strictness and high warmth, which are related to authoritative parents [30]. Parenting practices such as reasoning and dialogue to limit the child’s incorrect behaviors are positively related to the parental warmth dimension, which is related to authoritative and indulgent parents [31]. Different empirical studies have tested the organization of different parental practices on the two main parental dimensions across different cultural contexts as United States [30] or Europe [32,33] (see Figure 2). In general, the main findings from these studies agreed on the organization of different parental practices on the two main parental dimensions. Furthermore, the analysis of the impact of parenting practices on child adjustment is quite accurate in examining them within the general context of the parenting style (see Darling and Steinberg, 1993) [11]. For example, monitoring was originally identified as a parenting practice related to parental efforts to watch over their children as a form of strict or firm control [34,35]. Overall, parental monitoring predicted a wide range of adjustment outcomes. Nevertheless, most of the relationships between monitoring and adjustment outcomes can be explained by children’s spontaneous disclosure of information to their parents (authoritative parenting), but not by parents’ tracking and surveillance efforts (authoritarian parenting) (see Stattin and Kerr, 2000; Kerr and Stattin, 2000) [36,37].

Empirical research on parental socialization, mainly in Anglo-Saxon cultural contexts with European-American middle-class families, indicates that authoritative parents produce children with better psychosocial development, compared to children from the other three types of families [3,6,11,15]. However, results of studies with ethnic minorities from the United States, for example, Chinese-Americans [45,46], Hispanic-Americans [47,48], or African-Americans [49,50], as well as studies in Arab societies [51,52], question the idea that authoritative parenting (parental strictness with warmth) is always the most appropriate parental socialization style. These studies show that the authoritarian style (parental strictness without warmth) is positively related to some indicators of psychosocial development in children and adolescents.

Likewise, a growing set of studies, carried out mainly with families from European [53,54] and Latin-American countries [55,56], suggests that the indulgent style (parental warmth without strictness) is associated with equal or even greater psychosocial development than the authoritative style [57,58,59,60]. Children with indulgent parents, compared to their peers in authoritative families, show equal or even better adjustment and competence indices on indicators of optimal psychosocial development, such as self-concept [53,54], emotional regulation [61], school competence [62], internalization of values [63], including self-transcendence (universalism and benevolence) and conservation (security, conformity, and tradition), or connectivity with the environment [64], and protection from problems such as substance use [65], alcohol consumption [66], personal maladjustment indicators [67], and emotional maladjustment, including nervousness and hostility [58], sexist attitudes [68], and cyber-aggression and cybervictimization [69].

The parental socialization literature shows discrepant results about the relationship between parenting styles and child development, depending on the ethnic, socioeconomic, or cultural context where parental socialization takes place [9,70,71,72,73]. Additionally, although the impact of parental socialization is known to be crucial for the child, once adolescence is over and it is necessary to have the skills demanded by the adult world [1,74,75], few studies have examined long-term parental socialization beyond adolescence. Most of these studies have been limited to young adults [58], or adolescents and older adults have been compared using different adjustment criteria [76]. Although developmental theorists have highlighted the importance of early socialization experiences on development well beyond adolescence [3,77], less is known about the links between parenting and developmental outcomes in adulthood [76]. This may be due to the many non-normative influences in adulthood or the time elapsed since the early socialization experiences [78]. Therefore, a crucial question is whether differences in development, in both adolescent and adult children, can be consistently related to parenting styles. In the present study, the relationship between parental socialization styles (i.e., indulgent, authoritative, authoritarian, and neglectful) and short-term and long-term psychosocial development in adolescents and adults (young, middle-aged, and older) was examined through a wide range of indicators (physical and family self-concept, nervousness, empathy, and internalization of social values of benevolence). Importantly, some previous parenting studies have examined these indicators of psychosocial development, but in isolation rather than simultaneously. Overall, these indicators have been identified as important for psychosocial development. Physical self-concept, which includes self-perceptions about physical appearance and physical performance, is positively related to physical fitness [79] and a protective factor against eating disorders [80] or food neophobia [81]. Family self-concept, which includes self-perceptions about feeling loved and appreciated by the family, is positively related to general self-esteem [82] or secure attachment [83]. Nervousness is an indicator of emotional instability that is linked to aggressive behaviors or alcohol consumption [84,85]. Empathy, which involves sharing someone else’s perceived emotion—“feeling with” another [86], is related to more prosocial behaviors and less aggressive behaviors [87]. The values of benevolence are part of the self-transcendence values, identified in the Swartz theoretical framework as being strongly connected to prosocial behaviors and empathy [88,89]. In particular, benevolence values might be more connected to empathy than the other nine social values proposed by Swartz [90]. Based on previous studies, children from indulgent families are expected to show equal or even better psychosocial development on all the indicators than children from authoritative families, whereas children from neglectful and authoritarian families are expected to obtain the worst adjustment on all the indicators.

## 2. Materials and Methods

### 2.1. Participants and Procedure

The present study involved 2150 adolescent and adult children (*M =* 35.63, *SD =* 20.67), 1262 women (57.7%) and 888 men (58.7%): Adolescents (*n* = 623, 361 women, 59.7%) from 12 to 18 years old (*M =* 16.57; *SD =* 1.72); young adults (*n* = 619, 366 women, 59.1%) from 19 to 35 years old (*M =* 23.68 *SD =* 3.87); middle-aged adults (*n* = 502, 322 women, 64.1%) from 36 to 59 years old (*M =* 48.30; *SD =* 6.32); and older adults (*n* = 406, 213 women, 52.5%) over 60 years old (*M =* 68.66: *SD =* 7.80). Applying an a priori power analysis [91,92], the minimum sample required was 2150 observations to detect a power of 0.89 (α = 0.05; 1 - β = 0.95), with a medium-low effect size, *f* = 0.17, estimated value with the ANOVAs on a univariate *F* test of the four parenting styles [15,93]. Following previous studies on socialization with adolescent children and adult children, a similar procedure was followed for data collection in this study [5,58,63]. In short, adolescents were recruited from high schools that were selected randomly from the complete list of high schools; young adults from university undergraduate courses; middle aged-adults from city council neighborhoods; and older adults from senior citizen centers that were selected randomly from the complete list of senior citizen centers. Data collection was conducted during the 2017–2018 and 2018–2019 academic years. All the participants in this study: (a) were Spanish, as were their parents and their four grandparents; (b) lived in nuclear families with two parents, a mother or female primary caregiver and a father or male primary caregiver; (c) participated voluntarily, both adolescents and adult children, with the prior consent of their parents to participate in the case of the adolescents; (d) filled out the self-report paper-and-pencil questionnaires. According to the Declaration of Helsinki, the protocol was approved by the Research Ethics Committee of the Program for the Promotion of Scientific Research, Technological Development, and Innovation of the Spanish Valencian Region, the public institution that supports the present research. This study was also approved by the College Research Ethics Committee (CREC) of the Nottingham Trent University (NTU, Nottingham, UK; Project No. 2017/90).

### 2.2. Measures

#### 2.2.1. Parental Socialization

The parental warmth dimension was measured with the 20 items on the Warmth/Affection Scale, originally developed by Rohner (1978) [94] and widely used in parenting studies [95]. This scale offers a reliable measure of the degree to which children experience involvement and affection from their parents (Examples of items: “Say nice things to me when I deserve them” and “Are really interested in what I do”), and it also has an adult version (Examples of items: “Said nice things to me when I deserved it” and “Were really interested in what I did”). An alpha coefficient of 0.945 was obtained. The parental strictness dimension was measured with the 13 items on the Parental Control Scale, originally developed by Rohner (1978) [94], and widely used in parenting studies [96]. This scale offers a reliable measure of the degree to which children experience control with firmness, demand, and severity by their parents (Examples of items: “Give me certain jobs to do and will not let me do anything else until I am done” and “Want to control whatever I do”), and it also has an adult version (Examples of items: “Gave me certain jobs to do and would not let me do anything else until I was done” and “Wanted to control whatever I did”). An alpha coefficient of 0.904 was obtained. Both parental measures use a Likert-type response scale that ranges from 1 “almost never true” to 4 “almost always true”. Higher scores on the two parental measures represent high parental warmth and strictness.

The four parenting styles were defined following the dichotomization procedure based on the median of the scores on each of the two parental dimensions (i.e., warmth and strictness) and examining them simultaneously: Authoritative families scored above the median on both parental dimensions, neglectful families scored below the median on both parental dimensions, indulgent families scored above the median on parental warmth and below the median on parental strictness, and authoritarian families scored below the median on parental warmth and above the median on parental strictness [5,15,46].

#### 2.2.2. Psychosocial Development

Physical and family self-concept. These two dimensions of self-concept were measured with the family and physical scales of the AF5 Self-Concept Questionnaire [97]. The AF5 is a widely validated questionnaire in samples of adolescents and adults [98,99,100] in several countries, such as Spain [99], Portugal [100], Brazil [101], Chile [102], China [103], or the United States [104]. The AF5 theoretical factorial structure (i.e. multidimensional) is invariant across Western and non-Western societies [101,103,104]. The physical component refers to the individual’s perception of his/her condition and physical appearance (Example item: “I like the way I look). An alpha coefficient of 0.782 was obtained. The family component refers to the individual’s perception of his/her integration, involvement, and participation in the family environment (Example item: “My family would help me with any type of problem”). An alpha coefficient of 0.816 was obtained. Both measures of self-concept, with six items each, use a Likert-type response scale ranging from 1 “Strong disagreement” to 99 “Strong agreement”. Higher scores on both measures indicate a higher sense of family and physical self-concept.

Nervousness. It was measured with the 8 items on the nervousness scale of the Psychosocial Maturity Questionnaire [5,58,105]. Nervousness refers to the lack of emotional stability and anxiety in situations in everyday life (Example item: “My mood changes easily”). The scale uses a Likert-type response format ranging from 1 “Strongly disagree” to 5 “Strongly agree”. Higher scores on this scale indicate a higher degree of nervousness. An alpha coefficient of 0.778 was obtained.

Empathy. It was measured with the 5 items on the empathy scale of the Psychosocial Maturity Questionnaire [5,58,105]. Empathy refers to understanding others and considering other views apart from one’s own (Example item: “I am sensitive to others’ feelings and needs”). An alpha coefficient of 0.672 was obtained. The scale uses a Likert-type response format ranging from 1 “Strongly disagree” to 5 “Strongly agree”. High scores on this scale indicate a high degree of empathy.

Internalization of social values. The benevolence values were measured with the 5 benevolence scale items on the Schwartz Value Inventory [89,106]. The values of benevolence refer to the care of family relationships and values such as forgiveness (Example item: “Forgiving (Willing to pardon others)”). An alpha coefficient of 0.740 was obtained. The scale uses a Likert-type response format ranging from 1 “Opposed to my values” to 99 “of supreme importance”. Scores on this scale indicate that a high priority is given to benevolence values.

### 2.3. Plan of Analysis

A MANOVA (multivariate factorial analysis of variance) (4 × 2 × 4) was performed on the five indicators of psychosocial development (physical and family self-concept, nervousness, empathy, and internalization of social values of benevolence), with parenting styles (authoritative, authoritarian, indulgent, and neglectful), sex (women vs. men), and age (adolescents, young adults, middle-aged adults, and older adults) as independent variables. Univariate *F* tests were applied to all the sources of variation where statistically significant differences were found on multivariate tests. The significant univariate results were followed by post-hoc Bonferroni comparisons for all the possible pairs of averages [5,107,108].

## 3. Results

### 3.1. Parenting Styles

Participants were distributed in the four family types (see Table 1). On parental warmth, children from indulgent (*M =* 73.47; *SD =* 4.63) and authoritative (*M =* 72.45, *SD =* 4.37) families scored higher than children from authoritarian (*M =* 55.00; *SD =* 10.04) and neglectful (*M =* 57.04; *SD =* 9.52) families. On parental strictness, the children from authoritative (*M =* 40.05, *SD =* 5.00) and authoritarian (*M =* 41.90; *SD =* 5.52) families scored higher than the children from indulgent (*M =* 28.35, *SD =* 5.57) and neglectful (*M =* 28.25, *SD =* 5.79). The results from the correlation analysis confirmed the orthogonal theoretical assumption; the correlation between the two parental dimensions (i.e., warmth and strictness) was modest, *r* = −0.268, *R*^2^ = 0.072, less than 8%, *p* < 0.001 (the range in the shared variance, i.e., *R*^2^, is from 0 to 1).

### 3.2. MANOVAs Multivariate Analysis

Statistically significant differences were obtained for the main effects of parenting style, Ʌ = 0.742, *F*(15, 5836.2) = 44.45, *p* < 0.001; sex, Ʌ = 0.881, *F*(5, 2114.0) = 57.34, *p* < 0.001; and age, Ʌ = 0.895, *F*(15, 5836.2), = 16.02, *p* < 0.001 (see Table 2). The interaction effects between parenting styles and sex also reached the level of statistical significance, Ʌ = 0.985, *F*(15, 5836.2) = 2.17, *p* = 0.006; as did the effects between parenting styles and age, Ʌ = 0.958, *F*(45, 10562.0) = 2.00, *p* < 0.001; and between sex and age, Ʌ = 0.987, *F*(15, 5836.2) = 1.87, *p* = 0.021.

### 3.3. Parenting Styles

**Figure 3:**
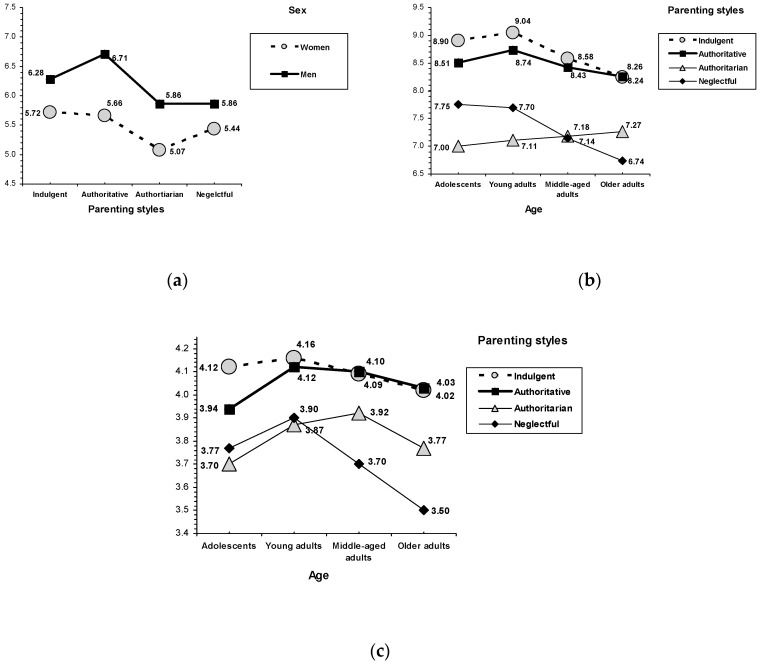
Interaction effects between parenting style and sex. (**a**) Physical self-concept. Interaction effects between parenting style and age. (**b**) Family self-concept, and (**c**) Empathy.

On the psychosocial adjustment criteria, the indulgent style was related to equal or even better scores than the authoritative style, whereas the lowest scores corresponded to the neglectful and authoritarian styles (see Table 3). (i) On physical self-concept, an interaction effect between parenting style and sex was found, *F*(3, 2118) = 2.82, *p* = 0.037 (see Figure 3). Although women always scored lower than men, a similar pattern was seen when examining parenting styles. In men, indulgent and authoritative styles were related to better physical self-concept than neglectful and authoritarian styles. In women, indulgent and authoritative styles were associated with the highest scores, whereas the authoritarian style was associated with the lowest scores. (ii) On family self-concept, an interaction effect between parenting style and age was found, *F*(9, 2118) = 5.49, *p* < 0.001 (see Figure 1). The indulgent style was related to higher scores than the authoritative style, a trend that was observed within the groups of teenagers and young adults. The authoritative style was associated with higher scores than the authoritarian and neglectful styles. Finally, the lowest scores on family self-concept were related to the authoritarian style, a trend that was observed in the groups of teenagers and young adults, but not in middle-aged adults, whereas in the group of older adults, the lowest scores corresponded to the neglectful style. (iii) In the case of nervousness, the children from indulgent and authoritative families obtained lower scores than their peers from authoritarian and neglectful households. (iv) For empathy, an interaction effect between parenting style and age was found, *F*(9, 2118) = 2.80, *p* = 0.003 (see Figure 1). Among the adolescents, the indulgent style was related to the highest scores on empathy, and the lowest scores corresponded to the authoritarian and neglectful parenting styles, with the authoritative style in an intermediate position. Similarly, in adult children, the indulgent and authoritative styles were associated with the highest scores (in all three groups), whereas the lowest scores corresponded to the authoritarian (in young adults) and neglectful (in young, middle-aged, and older adults) styles. (v) On the internalization of social values of benevolence, children with indulgent and authoritative parents reported higher scores than children from authoritarian and neglectful families.

### 3.4. Sex-and-Age-Related Differences

**Figure 4:**
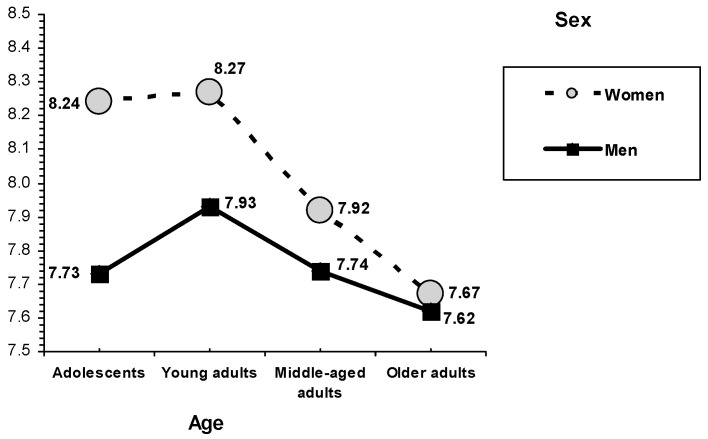
Interaction between sex and age. Family self-concept.

Statistically significant sex differences were found on the five psychosocial development criteria examined (see Table 4). On physical self-concept, men scored higher than women. On family self-concept, an interaction between sex and age was found, *F*(3, 2118) = 3.79, *p* = 0.010 (see Figure 4). Women indicated a higher family self-concept than men, a trend that was observed in adolescents and young adults and, to a lesser extent, in middle-aged adults (in older adults, men and women scored the same). On nervousness, empathy, and social values of benevolence, women scored higher than men.

Statistically significant differences in age were found on the five psychosocial development criteria examined (see Table 5). On physical self-concept, adolescents and young adults obtained higher scores than middle-aged adults, who, in turn, scored higher than older adults. On family self-concept, examining the sex profile by age (see Figure 4), the highest scores in women corresponded to the age group of adolescents and young adults, whereas the men who scored the highest were the young adults. On nervousness, adolescents and young adults scored higher than middle-aged and older adults. On empathy, young adults and middle-aged adults reported higher scores than adolescents and older adults. On priority given to benevolence values, middle-aged adults and older adults obtained the highest scores, whereas the lowest scores corresponded to adolescents (older adults exceeded young adults).

## 4. Discussion

This paper examined the relationship between parenting styles (indulgent, authoritative, authoritarian, and neglectful) and psychosocial development (physical and family self-concept, nervousness, empathy, and internalization of benevolence social values) in the short term in teenagers, and in the long term in adult children (young adults, middle-aged adults, and older adults). The measures of the parental dimensions (i.e., warmth and strictness) were modestly correlated, confirming the orthogonality theoretical assumption. The results of this study show a common pattern in the short and long term in the relationship between parenting styles and psychosocial development. The indulgent style (parental warmth without strictness) appears to be the optimal style, associated with equal or even better results than authoritative parenting (parental warmth with strictness), whereas the styles with a lack of parental warmth (authoritarian and neglectful) are related to low scores on the psychosocial development criteria examined.

In general, the results of the present study indicate that the indulgent and authoritative styles were related to better psychosocial adjustment indicators than the authoritarian and neglectful styles. In the case of physical self-concept, examining the profile of parenting styles by sex, we observed that, although men showed a higher physical self-concept than women, parenting styles characterized by warmth (indulgent and authoritative) were related to a higher physical self-concept in both men and women. With regard to family self-concept, examining the profile of parenting styles by age, we observed that the indulgent style was related to a higher family self-concept than the authoritative style (in adolescents and young adults), and the authoritative style was associated with a higher family self-concept than the authoritarian style. Regarding the parenting styles without parental warmth, children raised in authoritarian families obtained the lowest family self-concept scores (although this trend was only observed in adolescents and young adults). In the case of empathy, the profile of parenting styles by age revealed that the indulgent style was related to equal or better results (in adolescents), whereas neglectful and authoritarian parenting styles were related to less empathy (in middle-aged adult children and older adults, those who were raised by neglectful families indicated the lowest empathy). In relation to benevolence, parenting styles characterized by warmth (indulgent and authoritative) gave a higher priority to this social value than parenting styles characterized by lack of warmth (neglectful and authoritarian).

An important question that has been widely examined in the parenting literature for years has to do with the best parenting strategy to promote optimal psychosocial development [5,6,11]. The influence of parents on children is usually captured through parental warmth and strictness, both defined as orthogonal (i.e., unrelated) dimension [1,11]. The results from the present study confirm other previous research from studies mainly conducted in European [53,54,57] and Latin-American countries [9,55,56], where indulgent parenting (i.e., warmth without strictness) is related to equal or even better results than authoritative parenting (i.e., warmth with strictness), thus extending the evidence about the benefits of indulgent parenting to five important indicators of psychosocial development. Nevertheless, the present findings also contradict the commonly accepted idea about the benefits of parental strictness combined with parental warmth for child development, based mainly on results from studies conducted with middle-class European-American families [6,11,15]. In these studies, children and adolescents from authoritative families have greater psychosocial development on different indicators than their peers from the other households [1,4,24]. It seems that children from families characterized by warmth and strictness (i.e., authoritative parenting) do not always develop the best competence and adjustment in all cultural contexts. For example, some previous research in ethnic minorities from the United States [46,49] as well as from Asian and Arab countries [51,52], revealed some benefits related to authoritarian parenting (i.e., warmth without strictness). Therefore, a particularly pressing issue is the different impact of the parenting styles depending on the child’s cultural background [11,71].

Another main contributions of this study is that it provides empirical evidence about the consistent links between parenting and psychosocial development, not only in the short term in adolescent children, but also in the long term, beyond adolescence, when parental socialization has ended, using five indicators of key psychosocial development in adolescence and adult life: the individual’s perception of him/herself in the physical and family environment (physical and family self-concept), the individual’s emotional stability (captured with an indicator of mismatch, that is, nervousness), the ability to understand and understand others (empathy), and the internalization of social values (priority given to benevolence). Theorists on parental socialization point out that during the socialization process, parents exert their influence on children through practices of affection, dialogue, reasoning (parental warmth), and firm or restrictive practices (parental strictness), in order to achieve one of the main objectives of socialization: for the child to become a responsible adult [1,2,3]. The findings of the present study show that, in both adolescents and adults (young, middle-aged, and older), the differences in psychosocial development can be theoretically predicted by the parental socialization style. Of the few previous studies that examined the long-term impact of parental socialization, most of them focused on young adults [58], they were conducted in countries with an Anglo-Saxon culture, or they compared adolescents and older adults using different adjustment criteria [76]. Therefore, the present study allows a more exhaustive comparison by using five identical key psychosocial criteria and examining the impact of short-term and long-term socialization in three groups of adults. In addition, although it is not the main objective of the study, our results coincide with sex and age differences found in some previous studies [58,63,109].

Adolescence is frequently characterized as the developmental period in which the physical and social status of a child changes to that of an adult [110] (p. 112). Adolescents are not yet adults, and their parents act as socializing agents by using practices based on warmth and strictness. The present study showed important age-related differences in the developmental indicators examined in adolescents and adults, but also among young, middle-aged, and older adults, confirming some results from developmental studies about age-related differences across the life span [78,111]. In adolescence, but especially in adulthood, there are many influences (e.g., biological, personal, social and cultural) on development [57]. Despite these influences, at least in adolescence, previous parenting studies showed that differences in competence and adjustment among adolescents can be consistently related to parenting styles—in the same cultural context—[11,71]. Much less is known about what happens in adulthood. Nevertheless, as in some previous emergent studies, the main findings revealed that differences in competence and adjustment among adult children are also related to parenting. As in most of the previous studies with adult children [112,113], the present study follows a cross-sectional design, and so the results should be considered preliminary. Longitudinal evidence is more difficult to obtain for adult children, but the few longitudinal studies [114] have also revealed consistent links between parenting and adult development.

This study has strengths and limitations. The use of the four-typology parental socialization model provides a common framework with which to compare the results of research conducted in different countries around the world. The impact of parental socialization is examined beyond adolescence, with three groups of adult children and five psychosocial development criteria. Therefore, in addition to contributing to the current debate on the best parental socialization strategy, the findings of this study allow us to verify whether differences in psychosocial development between adults are also theoretically predictable based on parenting styles. As study limitations, it should be noted that the responses were provided by the adolescents and adult children [115], although there is evidence suggesting lower social desirability in children than in their parents [116]. Additionally, participants from the present study only include Spanish families and their adolescent and adult children. Therefore, future studies should extend these findings by examining parenting in immigrant families or ethnic minorities in Spanish society. In addition, the results of this study should be interpreted with caution, without inferring causal relationships or excluding possible third variables (especially considering the time elapsed for older adults), although these results are similar to those found in other studies with adolescents.

## 5. Conclusions

This study makes a valuable contribution to the current international debate about the suitability of parental socialization styles [72,73,117]. On the one hand, the findings of this work coincide with a set of studies, mainly carried out in countries in Europe [53,54] and Latin America [55,56], suggesting that the indulgent style is the optimal parental socialization style. However, the findings of the present study do not coincide with the classic studies mainly carried out with samples of middle-class European-American families [3,6,11,15]. The cultural context where socialization occurs seems to be decisive in the relationship between parental socialization styles and the children’s pattern of competence and adjustment. Future studies should examine the most appropriate parental strategy to socialize children considering families from different ethnic and cultural contexts [118,119,120,121], as well as different individual characteristics of the children [122,123].

## Figures and Tables

**Table 1:** Distribution of participants by parenting style.

	Total	Indulgent	Authoritative	Authoritarian	Neglectful
Warmth					
*Mean*	64.48	73.47	72.45	55.00	57.04
*SD*	1.52	4.63	4.37	10.04	9.52
Strictness					
*Mean*	34.69	28.35	40.05	41.90	28.25
*SD*	8.46	5.57	5.00	5.52	5.79
N (%)	2150 (100%)	628 (29.20%)	451 (21.00)	620 (28.80%)	451 (21.00%)

**Table 2:** Multivariate factorial analysis of variance (4^a^ × 2^b^ × 3^c^) for the indicators of psychosocial development (physical and family self-concept, nervousness, empathy, and internalization of social values).

Source of Variation	Λ	*F*	*df*_between_	*df*_error_	*p*
(A) Parenting Style ^a^	0.742	44.45	15	5836.2	<0.001
(B) Sex ^b^	0.881	57.34	5	2114.0	<0.001
(C) Age ^c^	0.895	16.02	15	5836.2	<0.001
A × B	0.985	2.17	15	5836.2	0.006
A × C	0.958	2.00	45	9459.5	<0.001
B × C	0.987	1.87	15	5836.2	0.021
A × B × C	0.974	1.23	45	9459.5	0.138

^a^*a*_1_, indulgent, *a*_2_, authoritative, *a*_3_, authoritarian, *a*_4_, neglectful; ^b^*b*_1_, male, *b*_2_, female; ^c^*c*_1_, adolescents, *c*_2_, young adults, *c*_3_, middle-aged adults, *c*_4_, older adults.

**Table 3:** Means, standard deviations (in parenthesis), *F* values, and Bonferroni’s test across parenting styles on the indicators of psychosocial development (physical and family self-concept, nervousness, empathy, and internalization of social values of benevolence).

Psychosocial Development	Indulgent	Authoritative	Authoritarian	Neglectful	*F*(1, 2118)	*p*
Physical self-concept	5.94 ^1^	6.15 ^1^	5.39 ^2^	5.61^2^	18.10	<0.001
	(1.74)	(1.79)	(1.83)	(1.68)		
Family self-concept	8.74 ^1^	8.51 ^2^	7.13 ^4^	7.41 ^3^	212.01	<0.001
	(0.95)	(1.04)	(1.57)	(1.50)		
Nervousness	2.20 ^2^	2.29 ^2^	2.56 ^1^	2.49 ^1^	38.06	<0.001
	(0.63)	(0.61)	(0.64)	(0.63)		
Empathy	4.11 ^1^	4.05 ^1^	3.81 ^2^	3.74 ^2^	55.32	<0.001
	(0.50)	(0.52)	(0.61)	(0.63)		
Social values	8.26 ^1^	8.20 ^1^	7.80 ^2^	7.67 ^2^	40.33	<0.001
of benevolence	(1.04)	(1.03)	(1.20)	(1.21)		

*Note:* Bonferroni test α = 0.05; 1 > 2 > 3 > 4.

**Table 4:** Means, standard deviations (in parenthesis), *F* values, and Bonferroni’s test between women and men on the indicators of psychosocial development (physical and family self-concept, nervousness, empathy, and internalization of social values of benevolence).

Psychosocial Development	Women	Men	*F*(1, 2118)	*p*
Physical self-concept	5.46	6.18	83.57	<0.001
	(1.76)	(1.75)		
Family self-concept	8.07	7.76	21.11	<0.001
	(1.44)	(1.51)		
Nervousness	4.04	3.78	104.42	<0.001
	(0.57)	(0.58)		
Empathy	2.44	2.31	22.13	<0.001
	(0.66)	(0.62)		
Social values	8.13	7.79	42.94	<0.001
of benevolence	(1.12)	(1.16)		

*Note:* Bonferroni test α = 0.05.

**Table 5:** Means, standard deviations (in parenthesis), *F* values, and Bonferroni’s test across age groups on the indicators of psychosocial development (physical and family self-concept, nervousness, empathy, and internalization of social values of benevolence).

Psychosocial Development	Adolescents	Young Adults	Middle-aged Adults	Older Adults	*F*(3, 2118)	*p*
Physical self-concept	6.13 ^1^	5.96 ^1^	5.54 ^2^	5.15 ^3^	33.72	<0.001
	(1.83)	(1.78)	(1.71)	(1.65)		
Family self-concept	8.03 ^a^	8.13 ^1^	7.86 ^2^	7.65 ^2,b^	13.83	<0.001
	(1.49)	(1.46)	(1.43)	(1.48)		
Nervousness	2.44 ^1^	2.40 ^1^	2.33 ^2^	2.34 ^2^	2.69	<0.001
	(0.64)	(0.65)	(0.65)	(0.64)		
Empathy	3.89 ^2,b^	4.01 ^1^	3.96 ^1,a^	3.84 ^2^	8.10	<0.001
	(0.57)	(0.55)	(0.58)	(0.64)		
Social values	7.78 ^2^	8.00 ^b^	8.19 ^1^	8.04 ^1,a^	12.19	<0.001
of benevolence	(1.23)	(1.09)	(1.05)	(1.18)		

*Note:* Bonferroni test α = 0.05; 1 > 2 > 3; a > b.
